# “Let’s face it… it’s futile”: Experiences of futility among nurses who provide care to patients with borderline personality disorder

**DOI:** 10.1192/j.eurpsy.2022.1704

**Published:** 2022-09-01

**Authors:** C. Papathanasiou, S. Stylianidis

**Affiliations:** Panteion University of Social and Political Sciences, Psychology, Athens, Greece

**Keywords:** mental health nursing, staff experiences, futility, borderline personality disorder

## Abstract

**Introduction:**

Research studies suggest that mental health nurses hold negative attitudes towards patients diagnosed with borderline personality disorder (BPD).

**Objectives:**

The aim of this study was to explore mental health nurses’ experiences and attitudes towards BPD patients in Greece, using a qualitative approach.

**Methods:**

Data were collected through two audio-recorded focus group discussions. The participants were twelve nurses who work in two General Hospital Psychiatric Units –one in Athens and one regional– and have direct clinical experience with BPD patients. The audio recordings were transcribed verbatim and analysed using thematic analysis in the context of grounded theory.

**Results:**

One overarching theme and three main themes were identified. The overarching theme that emerged was: “Futility”, which refers to feelings that the provision of nursing care to BPD patients is devoid of purpose and meaning. The main themes were: “Uncertainty”, which refers to the absence of valid causal explanations for mental disorders and on the perplexity of the BPD psychopathology; “Frustration”, which refers to challenges and barriers to providing care to BPD patients; “Unsupportiveness”, which refers to a complex mental health system, where there is a lack of guidance and goal orientation. Of significance are the nurses’ feelings of frustration and futility creating a sense of being burdened and leading to negative attitudes and behaviours towards BPD patients.

**Conclusions:**

Understanding treatment goals from the recovery model perspective and developing guidelines to help nurses revisit the concept of medical futility, may improve care to BPD patients.

**Disclosure:**

No significant relationships.

